# Supplementary material for: Interferon-Mediated Long Non-Coding RNA Response in Macrophages in the Context of HIV
Source: Int J Mol Sci. 2020 Oct 19;21(20):7741. doi: 10.3390/ijms21207741 (PMC7589721; doi:10.3390/ijms21207741)
Supplement: Supplementary file 1 [file ijms-21-07741-s001.pdf]

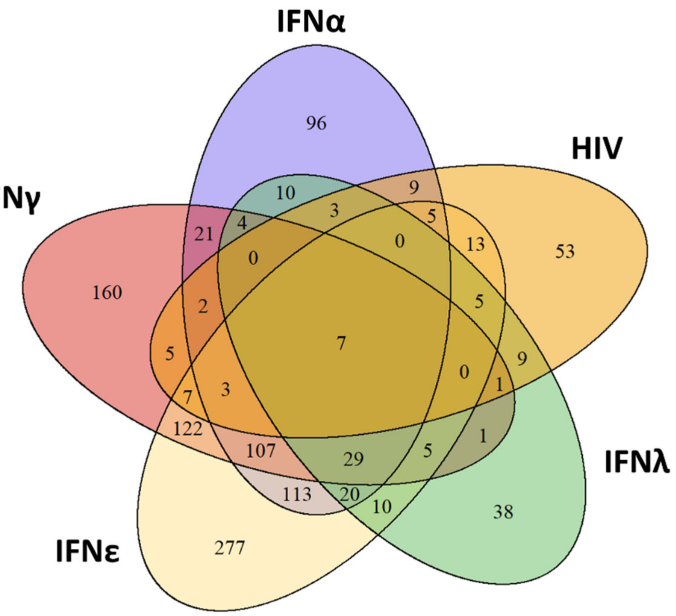

Figure 1. Quintuple Venn diagram showing the number of overlapping and non-overlapping differentially expressed non-coding genes in the interferon stimulated or HIV-1-BaL infected monocyte-derived macrophage conditions.

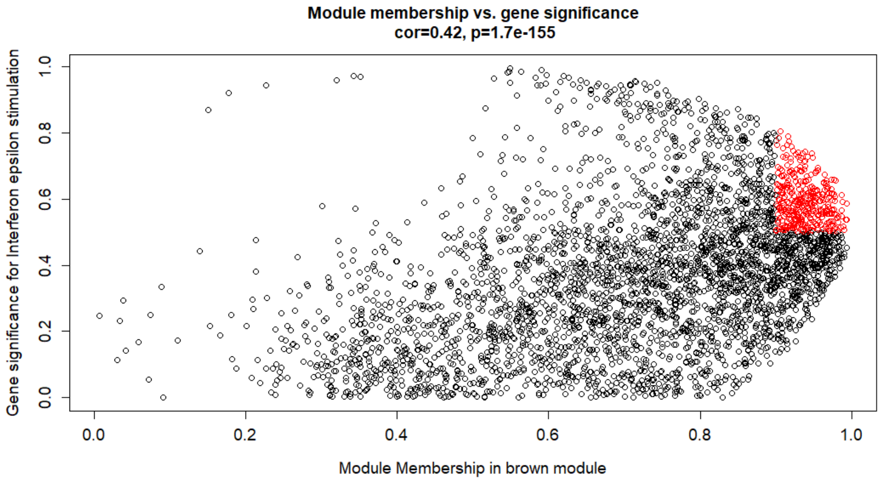

Figure 2. Scatterplot showing for each individual gene within the brown module the gene significance for IFN-ε stimulation vs it's module membership. Genes that are considered hub genes are depicted in red.

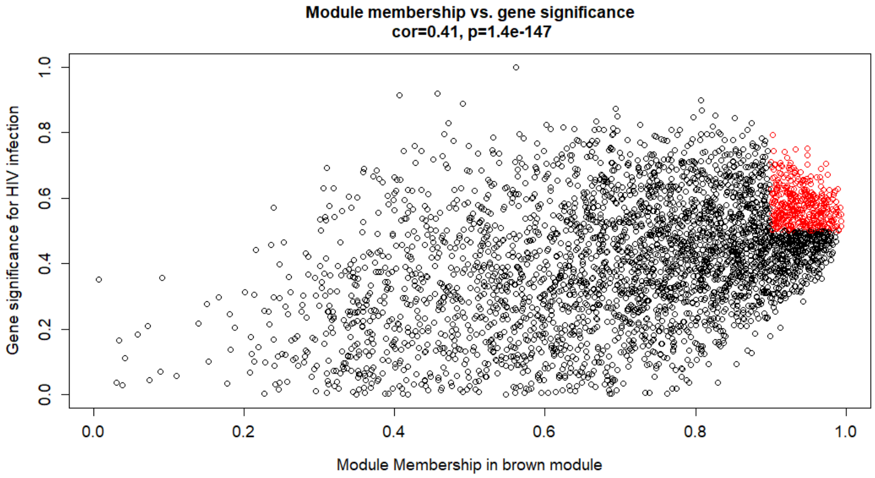

Figure 3. Scatterplot showing for each individual gene within the brown module the gene significance for HIV infection vs it's module membership. Genes that are considered hub genes are depicted in red.

Table 1. Five most significantly enriched Gene Ontology Biological Process (GO_BP) terms for the differential expressed genes upon interferon stimulation

Condition	ID	Biological Process	Number of Genes	Percentage of genes in annotation	FDR	Padj
IFNα	GO:0051707	response to other organism	283	17.64%	5.53E-32	8.72E-32
	GO:0043207	response to external biotic stimulus	283	17.62%	5.53E-32	1.11E-31
	GO:0009607	response to biotic stimulus	285	17.45%	1.19E-31	3.58E-31
	GO:0034097	response to cytokine	242	18.80%	4.15E-31	1.66E-30
	GO:0006952	defense response	305	16.49%	8.61E-30	4.31E-29
IFNγ	GO:0034097	response to cytokine	274	21.29%	1.64E-29	1.99E-29
	GO:0006952	defense response	352	19.03%	1.64E-29	3.27E-29
	GO:0034341	response to interferon-gamma	89	42.79%	5.21E-29	1.56E-28
	GO:0009607	response to biotic stimulus	320	19.60%	7.12E-29	2.85E-28
	GO:0071345	cellular response to cytokine stimulus	256	21.55%	1.41E-28	7.04E-28
IFNϵ	GO:0034097	response to cytokine	377	29.29%	2.25E-45	2.25E-45
	GO:0071345	cellular response to cytokine stimulus	351	29.55%	7.36E-43	1.47E-42
	GO:0006952	defense response	477	25.78%	1.78E-42	5.35E-42
	GO:0009607	response to biotic stimulus	419	25.66%	1.11E-35	4.43E-35
	GO:0051707	response to other organism	412	25.69%	3.71E-35	1.86E-34
IFNλ	GO:0006952	defense response	167	9.03%	1.76E-43	1.76E-43
	GO:0051707	response to other organism	151	9.41%	8.70E-41	2.23E-40
	GO:0043207	response to external biotic stimulus	151	9.40%	8.70E-41	2.61E-40
	GO:0009607	response to biotic stimulus	151	9.25%	5.35E-40	2.14E-39
	GO:0051607	defense response to virus	62	23.75%	8.07E-36	4.08E-35
HIV infection	GO:0019221	cytokine-mediated signaling pathway	68	8.35%	2.48E-22	2.48E-22
	GO:0034097	response to cytokine	81	6.29%	6.82E-20	1.36E-19
	GO:0071345	cellular response to cytokine stimulus	76	6.40%	8.15E-19	2.45E-18
	GO:0051607	defense response to virus	36	13.79%	1.15E-16	4.61E-16
	GO:0009615	response to virus	39	11.14%	3.65E-15	1.83E-14

Table 2. Five most significantly enriched KEGG pathways for the differential expressed genes upon interferon stimulation

Condition	ID	KEGG Pathway	Number of Genes	Percentage of genes in annotation	FDR	Padj
IFNalpha	83054	Cell cycle	32	25.81%	5.27E-03	1.16E-02
	172846	Staphylococcus aureus infection	20	35.71%	5.27E-03	1.27E-02
	83051	Cytokine-cytokine receptor interaction	53	19.63%	5.27E-03	1.58E-02
	217173	Influenza A	39	22.54%	5.45E-03	2.18E-02
	122191	NOD-like receptor signaling pathway	38	22.35%	8.11E-03	4.06E-02
IFNgamma	172846	Staphylococcus aureus infection	31	55.36%	2.50E-10	2.50E-10
	217173	Influenza A	51	29.48%	5.38E-06	1.08E-05
	200309	Rheumatoid arthritis	33	36.67%	2.10E-05	6.31E-05
	128760	Intestinal immune network for IgA production	23	46.94%	2.44E-05	1.15E-04
	83123	Allograft rejection	20	52.63%	2.44E-05	1.22E-04
IFNepsilon	83051	Cytokine-cytokine receptor interaction	91	33.70%	1.00E-08	1.00E-08
	812256	TNF signaling pathway	45	41.67%	6.51E-06	1.30E-05
	122191	NOD-like receptor signaling pathway	60	35.29%	1.04E-05	3.12E-05
	99051	Chemokine signaling pathway	61	33.52%	5.94E-05	2.38E-04
	634527	NF-kappa B signaling pathway	39	41.05%	8.81E-05	4.41E-04
IFNgamma	217173	Influenza A	28	16.18%	6.17E-08	6.17E-08
	83051	Cytokine-cytokine receptor interaction	32	11.85%	3.50E-06	7.00E-06
	377873	Herpes simplex infection	25	13.51%	2.29E-05	6.87E-05
	122191	NOD-like receptor signaling pathway	22	12.94%	4.71E-04	1.88E-03
	99051	Chemokine signaling pathway	22	12.09%	1.34E-03	6.69E-03
HIV infection	83051	Cytokine-cytokine receptor interaction	30	11.11%	1.03E-09	1.03E-09
	217173	Influenza A	21	12.14%	1.44E-06	2.87E-06
	122191	NOD-like receptor signaling pathway	16	9.41%	1.07E-02	3.21E-02

Table 3. Primers used for quantitative PCR.

Gene	Forward primer (5'-3')	Reverse primer (5'-3')	Reference
ACTB	TTCCTTCCTGGGCATGGAGT	TACAGGTCTTTGCGGATGTC	
B2M	AGATGAGTATGCCTGCCGTGTGAA	TGCTGCTTACATGTCTCGATCCCA	
DANCR	GCCACTATGTAGCGGGTTTC	ACCTGCGCTAAGAACTGAGG	Zhang *et al.* (1)
FIRRE	CTGTGACCTCGCTTCACTTCT	GTGGCAAAGAGCAGAAGATAGA	Lu *et al.* (2)
GAPDH	AGCCTCAAGATCATCAGCAATGCC	TGTGGTCATGAGTCCTTCCACGAT	
IFIT2	ACTGCAACCATGAGTGAGAAC	GCCTCGTTTTGCCCTTTGAG	Mariotti *et al*. (3)
MX1	CTGTAAATCTCTGCCCCTGTTAG	TCGTGTCGGAGTCTGGTAAAC	Mariotti *et al*. (3)
NRIR	CTGTCTCATCCAGTGAAGAC	TTGCAGTGAGCCAATATCGC	Mariotti *et al*. (3)
PLOD1	CAACAACAAGGACAACCGCATCCA	GAATTTGTGCCACTCCCGCTCAAA	
RP11-177F15.1	CTCCTGTGGGTTAGAACCATTTA	AAAGCTGAAGGTCCCAGAATAG	
RP3-477O4.14	GATCCGTCTTGCTCTCAGTTAG	AATGAGGCTTTGGCGTTAGA	
RSAD2	AGCATCGTGAGCAATGGAAG	GAAAGCGACTCTATAATCCC	Mariotti *et al*. (3)
TBP	CAAGCGGTTTGCTGCGGTAATCAT	TGCCAGTCTGGACTGTTCTTCACT	
YWHAZ	ACTTTTGGTACATTGTGGCTTCAA	CCGCCAGGACAAACCAGTAT	

1. Zhang J, Tao Z, Wang Y. Long noncoding RNA DANCR regulates the proliferation and osteogenic differentiation of human bone-derived marrow mesenchymal stem cells via the p38 MAPK pathway. International journal of molecular medicine. 2018;41(1):213-9.
2. Lu Y, Liu X, Xie M, Liu M, Ye M, Li M, et al. The NF-kappaB-Responsive Long Noncoding RNA FIRRE Regulates Posttranscriptional Regulation of Inflammatory Gene Expression through Interacting with hnRNPU. J Immunol. 2017;199(10):3571-82.
3. Mariotti B, Servaas NH, Rossato M, Tamassia N, Cassatella MA, Cossu M, et al. The Long Non-coding RNA NRIR Drives IFN-Response in Monocytes: Implication for Systemic Sclerosis. Frontiers in immunology. 2019;10:100.
